# Supplementary material for: Chromothripsis during telomere crisis is independent of NHEJ, and consistent with a replicative origin
Source: Genome Res. 2019 May;29(5):737–49. doi: 10.1101/gr.240705.118 (PMC6499312; doi:10.1101/gr.240705.118)
Supplement: Supplemental Material [file supp_gr.240705.118_Supplemental_file_1.zip › contigs/annotated_contigs/DB68/contig.2.DB68_length_672_mean_cov_6.56994047619.docx]

**DB68_length_672_mean_cov_6.56994047619**

```
CTCC|CCCTTCTCCCCTCCCCCTCCCCCGCTTCCTTCCCCTCCCCATCCTCCTCCCCCTCCTCC|CCCTCTTACTCCTCCTCC|CCTCC
     >chr2:82542503-82542562 + E=1e-08 p=0e+00                                    >chr
TCTTCCTCTTCCCCCTCCCACTTCCCCTCTCCCTCCTTCTCCTTCCTCCACCTCCTCCCTCCCTCTTCCTCCCCCCCCCCTCCTCCTCC
8:124219242-124219833 + E=0e+00
ACTTCCCCTCCCCATCCTTCCCCCTTCTCCTCCATCACTCTCTCTCCTCTTCCCCTCCAGCTTCACCTGGCTCTGCGGACACGTGCACC

GGAACATCCTCTCCAAGTTCACAGGCCAGGCGGTGGAGCTGTTTGACGAGGAGTTCCGCCACCTCTACGCCTCCTCCAAGCCTGTGATG

GGCCTGAAGTCCCCGCGGCTGGTCGCCCCCGTCCCGCCCGGAGCAGCCCCGGCCAATGGCCGCCTTAGCAGCAGCAGTGGCTCCGCCAG

TGACCGCACGTCCTCCAACCCCTTCAGCGGCCGCTCGGCAGGCAGCCACCCCGGTACCCGAAGTGTGTCCGCGTCTTCAGGGCCCTGTA

GCCCCGCGGCCCCACACCCGCCTCCACCGCCCCGGTTCCAGCCCCACCAAGGCCCTTGGGGAGCCCCGAGTCCCCAGGCCCACCTCTCC

CCGCGGCCCCACGACGGCCCGCCCGCCGCTGTCTACAGCAACCTGGGGGCCT
```
